# Association of Saccade Duration and Saccade Acceleration/Deceleration Asymmetry during Visually Guided Saccade in Schizophrenia Patients

**DOI:** 10.1371/journal.pone.0097308

**Published:** 2014-05-16

**Authors:** Hong Cui, Xiao-hui Liu, Ke-yong Wang, Chun-yan Zhu, Chen Wang, Xin-hui Xie

**Affiliations:** 1 Division of Medical Psychology, Chinese PLA General Hospital and Medical School PLA, Beijing, China; 2 Department of Psychiatry, Anhui Mental Health Center, Hefei, Anhui, China; 3 Department of Medical Psychology, Anhui Medical University, Hefei, Anhui, China; Institute of Psychology, Chinese Academy of Sciences, China

## Abstract

**Objective:**

To examine the difference between schizophrenia patients and normal controls on velocity and acceleration of saccade, by using the basic visually guided saccade (VGS) paradigm.

**Methods:**

Eighteen schizophrenia outpatients and fourteen normal controls participated in the VGS task. Multiple indicators, including amplitude, duration, velocity, latency, accuracy rate, acceleration, and deceleration were analyzed. Asymmetric acceleration index (AAI) was introduced to describe the difference between peak acceleration and peak deceleration. The correlation coefficient (R_AD_) of AAI and duration was computed to examine the difference between schizophrenia patients and normal controls.

**Results:**

No significant difference between patients and normal controls was found on amplitude, duration, latency, and accuracy rate. However, R_AD_ values of schizophrenia patients were significantly lower than the control group.

**Conclusion:**

Compared to normal controls, association of saccade duration and saccade acceleration/deceleration asymmetry during visually guided saccade was lower in schizophrenia patients.

## Introduction

Since Diefendorf and Dodge first reported the eye tracking dysfunction (ETD) in patients with schizophrenia in 1908 [1], ample studies on ETD have been conducted. Because eye movement requires precise adjustment and coordination of multiple nervous systems, eye tracking is very sensitive to functional damage of the brain [2], and consequently, some eye tracking tests have been used to study ETD in schizophrenia patients. Using indicators of smooth pursuit eye movements, anti-saccadic eye movements, and exploratory eye movements, *etc.*, some important studies [3]–[6] demonstrated that schizophrenia patients and their relatives have ETD. Visually guided saccade (VGS) is the eye movement whereby the participant rolls his/her eyeballs toward a visual transient, or stimulus [7]. As saccades have short durations (even the longest saccades last only about 100 ms, less than the response time of the visual system), there is no time for visual feedback, and accuracy depends on the internal monitoring of neural signals [8]. Baloh *et al.*, Van Opstal *et al.,* and other researchers showed that the velocity curve is not symmetric during the process of VGS, such that the value of acceleration phrase is larger than the deceleration phrase. In other words, the shape of the velocity curve is skewed to the left. Furthermore, this skewed characteristic is related to the amplitude and duration of the saccade [9], [10]. However, the mechanism of this phenomenon remains unknown. Nevertheless, due to direct or combined function of saccade-related brain regions, such as the prefrontal lobe, thalamus, superior colliculus, brainstem cerebellum, *etc.,* are widely found to be damaged in schizophrenia patients. Consequently, we hypothesize that patients with schizophrenia may show some differences in the correlation between velocity asymmetry and amplitude or duration. So, we focused on this phenomenon in schizophrenia to present some interesting discoveries in this preliminary report, which we hope will stimulate further research in this direction.

## Methods

### Ethics Statement

The study was approved by the Medical Research Ethics Committee of the PLA General Hospital. All participants were included in the study only if they had read the detailed study description and could correctly summarize all the procedures of the study. Participation was voluntary, and patients were allowed to reject or withdraw at any point. All participants enrolled in the study or their legal guardians signed written informed consent.

### Participants

Eighteen schizophrenic outpatients (9 males and 9 females) were recruited from the psychiatry department at PLA General Hospital. All patients met the DSM-IV diagnostic criteria for schizophrenia with paranoid (10 patients) and undifferentiated subtype (8 patients). All participants in our study did not have myopia, astigmatism, other ametropia, epilepsy, mental retardation, severe physical disease, or have been treated with electroconvulsive therapy within the past 6 months. All patients received antipsychotic medications during the study. We converted the antipsychotic doses into chlorpromazine-equivalent doses. A trained and experienced psychiatrist using the Positive and Negative Syndrome Scale (PANSS) [11] assessed the clinical symptoms of the patients. Fourteen healthy controls, with no personal history of psychiatric disease or family history of psychiatric illness, were recruited from the community by our advertisements. All participants were right-handed and were screened such that neurologic disease, metal implants, substance abuse, and people with a history of head injury were excluded. The schizophrenic patients and the healthy controls had no statistical differences in age and gender. The mean ages and standard deviations (SD) of the schizophrenic subjects and normal controls were 24.4±4.7 and 24.3±2.4 (Mann-Whitney *U* Test, *Z* = −0.267, *p* = 0.789). The years of education for schizophrenic participants and normal controls were 12.9±4.2 and 12.5±1.9 year (Mann-Whitney *U* Test, *Z* = −0.972, *p* = 0.331); the gender ratio was 6 male 8 female participants in control group, and 9 male 9 female in schizophrenia group (Mann-Whitney *U* Test, *Z* = −0.395, *p* = 0.693). Average scores on the PANSS were 70.7±11.7 in total score, 18.2±4.7 in PANSS positive score, and 19.5±2.6 in PANSS negative score for the schizophrenic subjects. The chloropromazine-equivalent dose in schizophrenia patients was 423.5±363.5 mg/day.

### Procedures

#### Stimulus

The experiment was created and presented using the Experiment Center Software (SensoMotoric Instruments GmbH, Germany). Stimuli were presented on a 22-inch LCD monitor (ThinkVision, Lenovo Ltd.). Each participant sat in a chair about 60 cm in front of a screen with his or her head fixed comfortably on a chinrest. An IVIEW X HI-SPEED eye tracker (SensoMotoric Instruments GmbH, Germany) was used to record participants' eye movements while they performed the tasks, and the sampling rate was set on 1250 Hz with the accuracy rate less than 1 degree and the spatial resolution about 0.01 degree. The task was a VGS task, in which a single black dot about 1 degree in sight was randomly displayed on gray background. The position of the dot was randomized, and the duration of a dot’s display was also randomized between 1000 ms and 1500 ms with no intervals between two dots. The participant was told to keep fixated on the black dots, and saccades were guided out as the positions of the dots changed. There were 10 practice trails and 100 formal trails, and the practice trails were not included in analyses. Saccade duration was detected with a velocity threshold of 40 degrees per second (°/s)and recorded automatically by computer. The following parameters were recorded during each saccade: amplitude (A), duration (D), latency, accuracy rate, peak velocity (V_max_), peak acceleration (P_acc_) and peak deceleration (P_dec_).

### Statistical Analysis

#### Descriptive analysis

To test whether VGS was significantly altered in schizophrenic patients, comparisons of each parameter between two groups was performed using independent student’s *t-*test and Mann-Whitney *U* tests as appropriate. Statistical significance was set at 0.05. Statistical analysis was carried out with SPSS for windows (SPSS 13.0, SPSS Inc, Chicago, IL, USA). The basic saccade parameters as duration, amplitude of saccade peak acceleration, and peak deceleration comparing between patients and normal controls were compared by independent student’s *t-*test. In addition, during eye saccades, three parameters (amplitude (A), V_max_ and duration (D)) were approximately described using a single equation
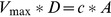
(1)where c is a dimensionless proportionality constant [10]. We performed this analysis for each participant and used the Spearman correlation coefficient between V_max_*D and A.

#### Special analysis

To examine the difference between schizophrenia patients and controls, a method with a specified index was used. First, because acceleration is sensitive to changes in velocity, we created an asymmetric acceleration index (AAI) shown in the following equation:
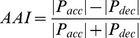
(2)


“P_acc_” stands for peak acceleration of saccade velocity curve, “P_dec_” stands for peak deceleration of saccade velocity curve. AAI describes the difference of slopes of acceleration phase and deceleration phase of saccade velocity curve by using the peak acceleration and peak deceleration. The AAI value of each saccade can be calculated easily. Second, correlations between AAI and duration were used to build another index R_AD_, which is the Spearman correlation coefficient between AAI and the duration. We also calculated the correlation coefficient of AAI and duration (R_AD_), amplitude (R_AA_), latency (R_AL_). R_AD_s, R_AA_s, and R_AL_s were calculated for each participant. Third, we examined the differences in R_AD_ and the relationship between R_AD_ and other parameters (gender, age, PANSS scores, *etc.*) in schizophrenic patients and the control group.

## Results

### Descriptive Analysis

**Figure 1:**
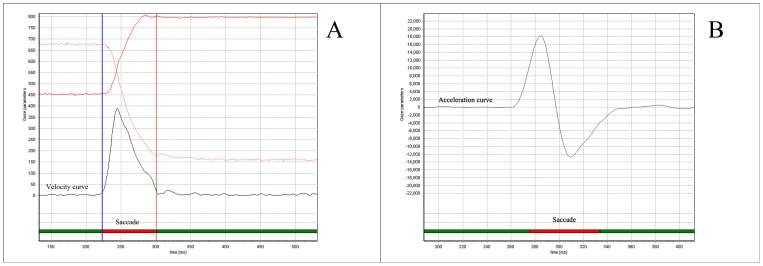
A: A typical velocity curve during one saccade. B: A typical acceleration curve during one saccade. Data of velocity curve and acceleration curve was extracted from one saccade of a normal control participant.

**Figure 2:**
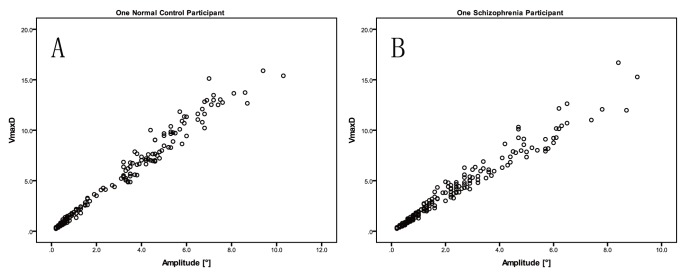
Scatter plots of V_max_*D and A. V_max_: Peak velocity; D: Saccade duration; A: Saccade Amplitude. Figure 2A: A normal control participant; Figure 2B: A schizophrenia patient.

The recorded saccade velocity profiles are asymmetrical, with a high acceleration phase and a low deceleration phase. A typical velocity curve during one saccade and a typical acceleration curve are presented in Figure 1. Figure 2 shows the scatter plot of V_max_*D versus A of two subjects (a healthy control and a schizophrenia patient). The basic saccade parameters as duration, amplitude of saccade peak acceleration, and peak deceleration between patients and normal controls were as follows: the differences in maximum velocity, and peak deceleration between the two groups were significant; the differences in latency, duration, amplitude, and peak acceleration between the two groups were not significant (Table 1). In the next analysis of equation-1, the Spearman correlation coefficient between V_max_*D and A for each participant was larger than 0.97, which means the association between these two factors is tight.

**Table 1:** Basic parameters of eye movements between normal control and schizophrenia group.

	Normal Control Group	Schizophrenia Group		
	Mean±SD	Mean±SD	*t*	*p*
Duration [ms]	45.83±26.35	44.93±24.86	1.362	0.173
Amplitude [°]	5.29±5.77	5.08±5.17	1.491	0.136
V_max_ [°/s]	164.94±120.04	174.08±127.78	−2.857	0.004*
P_acc_ [°/s^2^]	6706.89±5578.34	6938.54±5716.79	−1.581	0.114
P_dec_ [°/s^2^]	−4888.05±5573.44	−5691.06±7528.96	4.590	<0.001**

*p<0.005,

**p<0.001.

SD - Standard Deviation; V_max_ - Maximum Velocity; P_acc_ - Peak Acceleration; P_dec_ - Peak Deceleration.

### Special Analysis

**Figure 3:**
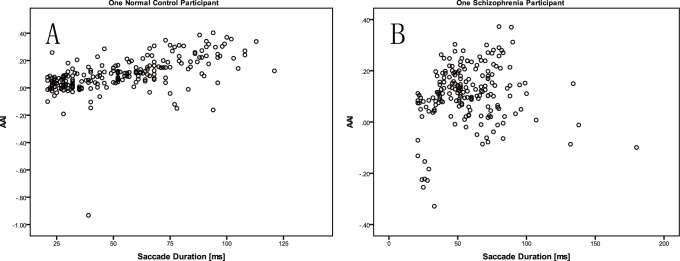
Scatter plots of AAI and D. AAI: Asymmetric acceleration index; D: Saccade duration. Figure 3A: A normal control participate; Figure 3B: A schizophrenia patient.

**Figure 4:**
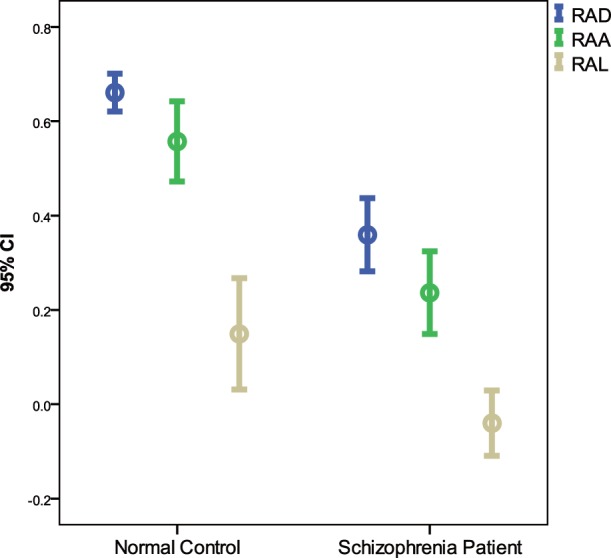
95% confidence interval (CI) plots of R_AD_, R_AA_ and R_AL_. R_AD_: The correlation coefficient of Asymmetric acceleration index (AAI) and saccade duration; R_AA_: The correlation coefficient of AAI and saccade amplitude; R_AL_: The correlation coefficient of AAI and saccade latency.

**Figure 5:**
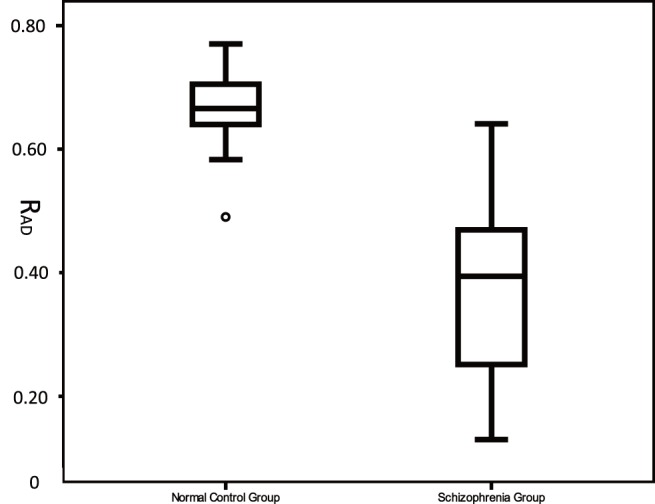
A boxplot of R_AD_. R_AD_: The correlation coefficient of Asymmetric acceleration index (AAI) and saccade duration. The R_AD_ shows significant difference between the schizophrenia and the normal control group (Mann-Whitney *U* test, *Z* = –4.521, *p*<0.00001).

A scatter plot of AAI versus duration from one participant from the control group is presented in Figure 3A, and a scatter plot of AAI versus duration for one participant from schizophrenia group is presented in Figure 3B. 95% confidence interval (CI) plots of R_AD_, R_AA_ and R_AL_ were presented in Figure 4, the 95% CI of R_AD_ of normal subjects was the shortest. A boxplot of R_AD_ is presented in Figure 5, and the R_AD_ shows a significant difference between the schizophrenia group and the control group (Mann-Whitney *U* test, *Z* = −4.521, *p*<0.00001). The correlations between the R_AD_ values and other factors are shown in Table 2.

**Table 2:** The Spearman correlation coefficients between R_AD_ value and other factors.

	Sex	Age	Accuracy	PANSS Total score	PANSS Positive score	PANSS Negative score	Antipsychotic medication dose*
**The Normal Control Group**							
**R_AD_**	−0.072	0.235	0.296	–	–	–	–
**The Schizophrenia Group**							
**R_AD_**	−0.139	−0.295	−0.142	−0.178	−0.259	0.007	−0.119

All correlation coefficients in the table did not reach the statistic significant.

*In chloropromazine-equivalent dose.

## Discussion

To our knowledge, the current research is the first psychiatric investigation on the association of the correlation between peak acceleration and deceleration and duration in VGS. This association in schizophrenia patients was significantly lower than it was in normal healthy people.

Velocity asymmetry of VGS was first described in detail by Baloh *et al.,* in which they described that in VGS, the change in velocity is not symmetric because velocity accelerates fast in the initial stage of saccade and decelerates slowly. In other words, the shape of the velocity curve skews to the left [9] (a typical velocity curve is shown in Figure 1A), and this asymmetric bias increases with the increase of the saccade’s amplitude. Van Opstal *et al.* demonstrated that the asymmetry associated with saccade duration is more fundamental than the correlation between asymmetry and amplitude [10], and that the acceleration, especially peak acceleration, is more sensitive to the change of velocity (a typical acceleration curve is shown in Figure 1B). Therefore, in this study, we chose the gap between the peak acceleration and peak deceleration and the duration of saccade, rather than the amplitude, to build R_AD_. Besides the ease of calculation, this simplified calculation method may have an extra advantage in that it is convenient in clinical settings. We choose to use the Spearman correlation coefficient to build R_AD_ because it was unknown whether or not the relationship between AAI and duration is linear [10]. It is noteworthy that there were some statistical differences on maximum velocity (V_max_) and peak deceleration (P_dec_) between two groups, but of the relationship between the maximum velocity (V_max_), amplitude (A), and duration (D), each subject showed an extremely high correlation between V_max_*D and A (r >0.97), and our result was consistent with that found by Van Osptal *et al.* [10]. That is, if the saccade is analogous to driving from point A to point B, our results may imply that schizophrenia patients can choose a correct maximum velocity according to the duration or length of the journey, just like normal controls, but they cannot choose an appropriate acceleration and deceleration. R_AD_ was an indirect indicator, and it can distinguished schizophrenia from normal population well. In this study, if the boundary value of R_AD_ was set at 0.55, only one schizophrenia patient was higher than this value, versus just one normal participant was lower than this value.

Velocity asymmetry and the relationship between this bias and the duration of saccade have been reported and repeatedly confirmed for decades, but the physiological meaning and the mechanism of the phenomenon are not yet clearly explained, and no relevant hypotheses have been proposed. Although our current understanding of the phenomenon is limited to the speculation that it may be associated with several cortical regions including the thalamus, superior colliculus, cerebellum, and brainstem, it does not prevent us from applying the phenomenon as an effective tool to study the brain damage of schizophrenia patients. Some researchers have shown that cerebral cortex structures in VGS mainly involve frontal eye field (FEF), supplementary eye field (SEF), pre-SEF, the dorsolateral prefrontal cortex (DLPFC), and the parietal eye fields (PEF) [12]. The complex relationship between these regions is still not clear, and some other studies have roughly sketched out some VGS-related circuits between these structures. The superior colliculus (SC) is located in the center of these circuits, and the DLPFC, posterior parietal cortex (PPC), prefrontal lobe, and parietal lobe project directly to the SC. To make a saccade to an object purposefully, the SC receives convergent inputs from cerebral cortical areas and the basal ganglia. The cortical inputs, in which the basal ganglia play another crucial role must select appropriate signals [13]. While the brainstem oculomotor nucleus in the subcortical structures, which is under the direct superior colliculus’s regulation, mainly participate in VGS [14], [15], the cerebellum is also involved in the control of VGS [16]. Many studies have shown that the prefrontal lobe, thalamus, cerebellum, and brainstem of schizophrenia patients are damaged by the disease [17], [18]. In addition studies of neurological soft signs suggest that the “cerebello-thalamo-prefrontal” brain network of schizophrenia patients is damaged [19]. Therefore, on the one hand, the fact that the confidence interval of R_AD_ in schizophrenia patients is significantly wider than normal control group maybe due to the pathological heterogeneity of schizophrenia. On the other hand, the significant reduction of R_AD_ in patients with schizophrenia is understandable and it deserves our attention. As the R_AD_s of the two groups were significantly different and the overlap between the two groups was small (Figure 4), we speculate that R_AD_ may have the potential to be a biological indicator of schizophrenia.

## Conclusion

Employing the basic task of VGS in schizophrenic patients and normal controls, our study investigated the correlation between the gap of peak acceleration and peak deceleration and the duration of saccade. The results indicate that R_AD_s are significantly lower in the schizophrenia group than in the normal control group. Our findings may suggest that R_AD_ has the potential to be a biological indicator of schizophrenia, and further studies need to be conducted in the future to test the viability of the diagnostic value of our findings.
